# Total ileocolic intussusception with rectal prolapse presenting in an adult: a case report and review of the literature

**DOI:** 10.1186/1749-7922-8-37

**Published:** 2013-09-23

**Authors:** James Frydman, Offir Ben-Ishay, Yoram Kluger

**Affiliations:** 1 Department of General Surgery, Rambam Health Care Campus, POB 9602, Haifa 31096, Israel

**Keywords:** Adult intussusception, Rectal prolapse, Zygosis

## Abstract

**Introduction:**

Intussusception is rarely encountered in adults, accounting for just 5% of all occurrences and 1% of bowel obstructions. In up to 90% of episodes of adult intussusceptions, operative intervention is required secondary to pathological lead points. Prior to the current report, only three cases of total ileocolic intussusception with rectal prolapse in adults have been described in the world literature, making it an important contribution to surgical knowledge. In addition to a discussion of disease etiology, this review outlines sound diagnostic and therapeutic principles in the successful management of this rare emergent surgical condition.

**Case presentation and literature review:**

In this case report, we will present a rare case of total ileocolic intussusception with rectal prolapse in a 22 year-old female without antecedent history. She had both a lead point in the cecum, as well as a highly mobile, intraperitoneal colon. Lead points have been found in only half of the reported cases, including this one. In addition, colonic laxity may enable this phenomenon, being attributed to the loss of *zygosis* during the embryological period, in which there is persistence of the ascending and descending mesocolons and lack of apposition to the retroperitoneum. The diagnostic work-up, operative strategy and pathological findings are discussed. The three previous cases reported in the English-language medical literature were reviewed.

**Conclusions:**

Adult intussusception, while uncommon, may be encountered in an acute surgical setting and optimal outcomes depend on a high index of suspicion and expeditious management. Embryological divergence may contribute to the even rarer variant of total ileocolic intussusception with rectal prolapse.

## Introduction

Intussusception in adults is rare, representing 1% of bowel obstructions and 5% of all intussusceptions [1]. Four categories are recognized, including entero-enteric (small bowel only), colo-colic (large bowel only), ileocolic (terminal ileum within ascending colon), and ileo-cecal (lead point is ileocecal valve) [2]. While intussusception in children is primary and benign, amenable to hydrostatic reduction in 80% of pediatric cases, it is secondary and pathological in up to 90% of adult presentations, requiring resection [2]. Diagnosis in adults is typically established in the operating room (OR) given the predominant symptoms of bowel obstruction. Underlying etiologies include polyps, carcinoma, Meckel's diverticulum, colonic diverticulum and strictures [1,2]. Total ileocolic intussusception with rectal prolapse in the adult is a rare emergent surgical condition with only four cases including the current report described in the world literature [3-5].

## Review

### Case presentation

**Figure 1:**
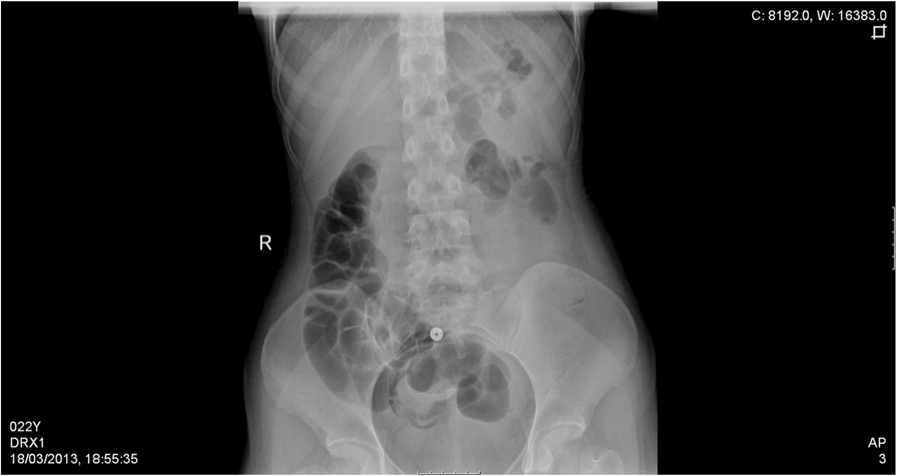
Plain abdominal supine radiograph revealing small bowel obstructive pattern with paucity of gas in colon.

**Figure 2:**
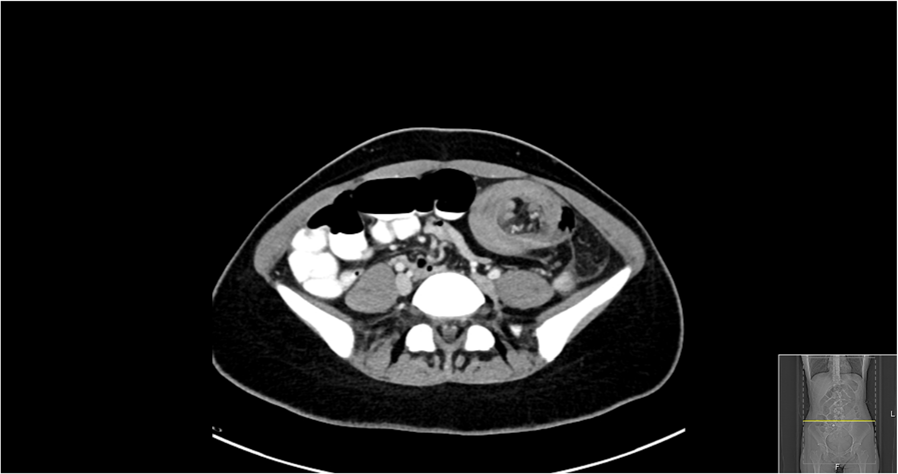
Axial section of abdominal CT revealing "target" sign of ileocolic intussusception in left abdomen.

**Figure 3:**
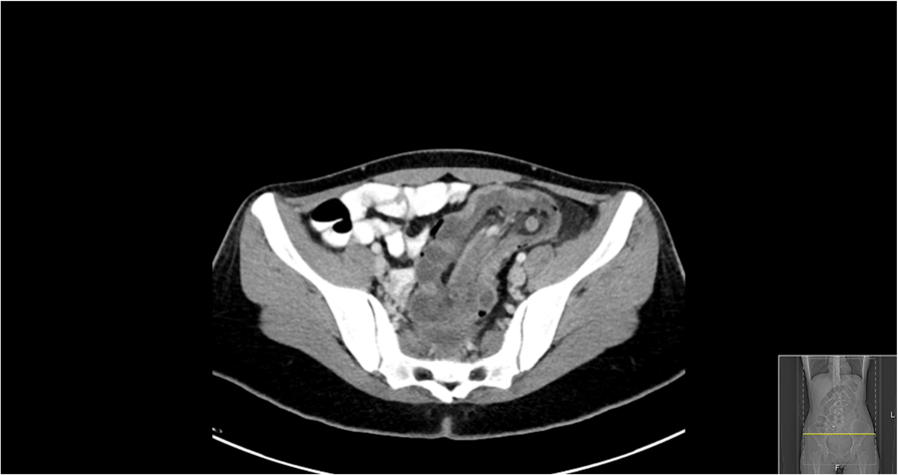
Axial section of pelvic CT revealing "sausage" sign of ileocolic intussusception to level of rectum.

A 22 year-old female with history significant only for anemia and no previous surgical history or family history of malignancy complained of abdominal pain and bleeding per rectum. At an outside facility, she was diagnosed with new-onset rectal prolapse which was reduced prior to presentation to our emergency department. On physical examination she was found to have abdominal tenderness without peritoneal signs and evidence of fresh blood on rectal exam without hemorrhoids or prolapse. Rigid proctoscopy confirmed bloody mucosal tissue without a clear source of hemorrhage and no evidence of ischemia. Laboratory values were unremarkable and abdominal films revealed a small bowel obstructive pattern with a paucity of identifiable gas in the colon. (Figure 1) Computed tomography (CT) scan of the abdomen and pelvis was subsequently performed with oral and intravenous contrast. An axial tomographic section taken from the abdomen demonstrates the "target" sign (Figure 2) of an extensive ileocolic intussusception, while a more distal section taken from the pelvis reveals the "sausage" sign (Figure 3) of the intussusception extending into the rectum.

**Figure 4:**
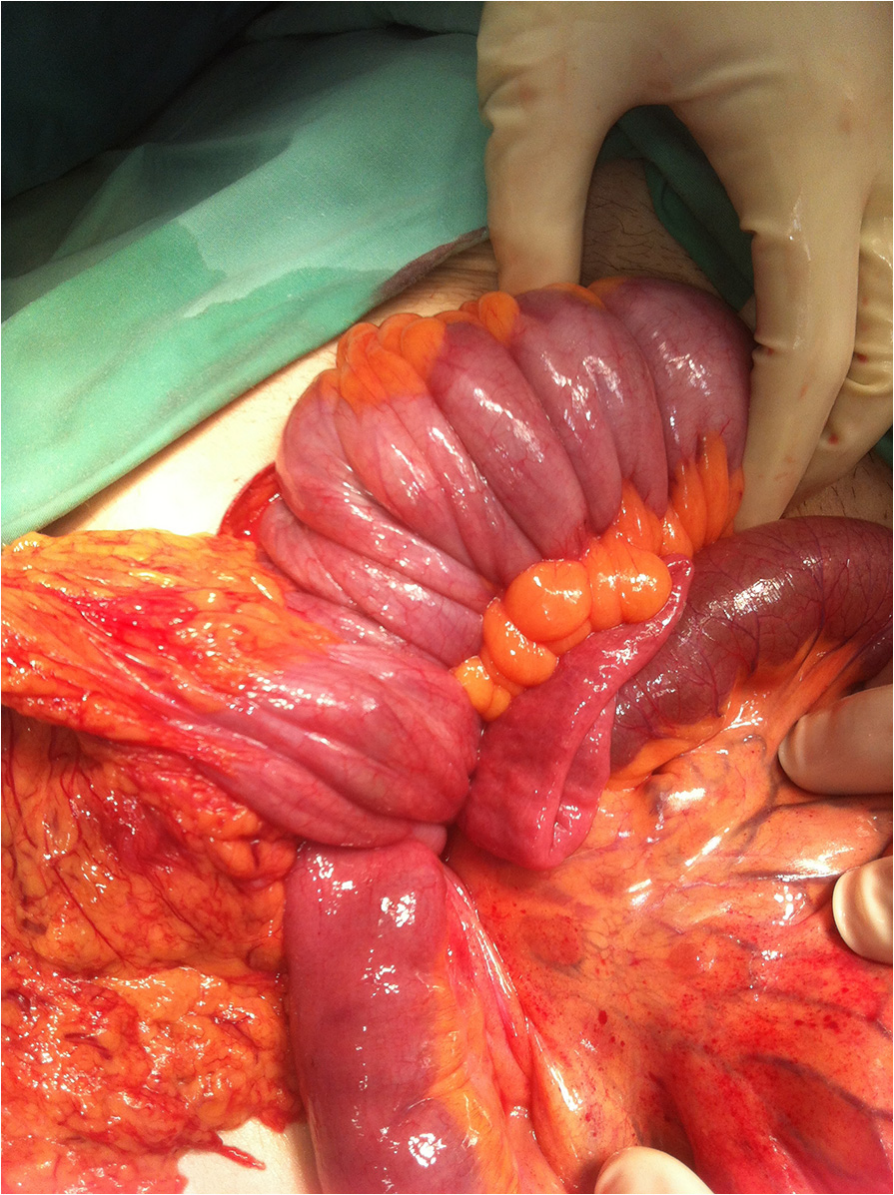
Intraoperative photo revealing total ileocolic intussusception to level of rectum.

The CT scan was concerning for total ileocolic intussusception to the level of the rectum with possible compromised bowel. The patient was brought to the OR for an urgent exploratory laparotomy. The distal small bowel was invaginated into the colon throughout its entire length and could be palpated in the upper rectum (Figure 4). The patient had a highly mobile colon with essentially absent flexures, without evidence of malrotation. We elected to proceed with distal to proximal reduction given the fact that a subtotal colectomy would have been mandated without this maneuver. The key technical points in performing this maneuver include localizing the distal aspect of the intussusception and careful milking proximally without undue manual pressure, in order to avoid inadvertant perforation. Success likely hinges on operative exploration early in the pathophysiological process. After successful reduction, a firm rubbery mass was palpated in the cecum. A formal right hemicolectomy was performed, given the risk of potential malignancy. Further exploration revealed a lipomatous mass in the wall of the proximal jejunum and segmental resection was performed. She was discharged home on post-operative day 10. Pathology revealed a fully resected 4 centimeter villous adenoma with foci of high grade dysplasia in the cecum. There was evidence of mucosal edema and lymphostasis in the adjacent colonic tissue. The small bowel specimen revealed ectopic pancreatic tissue. Given the pathological findings in this healthy 22 year-old female, the patient was referred for genetic counseling despite the negative family history, including testing for mutations and endoscopic screening.

## Discussion

While abdominal pain, nausea and emesis were the predominant presenting symptoms in 78% of patients in one large institutional series, the preoperative diagnosis was correct in only 33% in this same cohort [1].The widespread use of CT has helped to increase preoperative accuracy and can be virtually diagnostic of intussusception given its pathognomonic appearance. When the CT beam is parallel to the longitudinal axis, the intussusception will appear as a "sausage-shaped" mass. However, when the CT beam is perpendicular to the longitudinal axis, the intussusception will appear as a "target" mass. Furthermore, eccentric appearing mesenteric fat and vessels are often visible within the intussusception [6].

Intraoperative strategy in adult intussusception generally favors resection without reduction in adults given the high preponderence of tumoral lesions as lead points. As detailed in this case report, extensive intussusceptions involving the right colon may be selectively considered for careful distal to proximal manual reduction before definitive resection in order to avoid a more extensive resection or two-stage procedure [7]. This should not be attempted, however, when the bowel is ischemic, inflamed or friable as this could result in intraoperative perforation and loss of containment.

Interestingly, ileocolic intussusception presenting with rectal prolapse is exceedingly rare in adults, with only three cases previously reported in the English-language world literature (Table 1) [3-5]. David and colleagues describe ileosigmoid intussusception in a 50 year-old male with abdominal distention, constipation and a large mass protruding from the anus for three days. Prior to this presentation, he had diarrhea and a history of recurrent self-limiting episodes of intestinal obstruction. At surgery, he was found to have evidence of gangrenous changes in the intussuscepted ileum. A subtotal colectomy was performed though no pathological lead point on histology was demonstrated [5].

**Table 1:** Reported case of ileocolic intussusception with rectal prolapse in adults

**Author/Year**	**Journal**	**Age/Sex**	**Operation**	**Lead point**	***Zygosis***
Frydman (2013)	World Journal of Emergency Surgery	22, Female	Right Hemicolectomy	Yes, Cecal Villous Adenoma	No
Ongom (2013)	BMC Research Notes	32, Female	Right Hemicolectomy	None	No
Chen (2008)	Cases Journal	36, Male	Subtotal Colectomy	Yes, Ileocecal Submucosal Lipoma	Yes
David (2007)	Indian Journal of enterology	50, Male	Subtotal Colectomy	None	Yes

Chen and colleagues describe this presentation in a 36 year-old male who presented with an initial two month history of diarrhea followed by constipation and abdominal pain. While straining to defecate, a mass prolapsed from his anus and he presented for evaluation. His prolapsing mass was reduced but did not relieve his abdominal pain. Barium enema confirmed a filling defect in the sigmoid colon without proximal filling of the colon. At laparotomy, ileosigmoid intussusception was confirmed and could not be reduced, resulting in a subtotal colectomy. Pathology confirmed a 9x6x5 cm benign lipoma at the ileocecal junction [4].

Ongom and colleagues describe an ileocolic intussusception in a 32 year-old female who initially reported colicky abdominal pain and vomiting, associated with straining during defecation and incomplete evacuation of her rectum. Over the next two weeks prior to presentation, she noted continued colicky abdominal pain, bloody-mucoid discharge and a reducible mass protruding from her anus. On physical examination, an abdominal mass was palpated in the umbilical region and rectal mass noted 3cm proximal to the anal verge. Abdominal ultrasound confirmed the presumptive diagnosis of prolapsed intussusception with partial bowel obstruction. The mass was only able to be partially reduced in a distal to proximal direction and a subsequent right hemicolectomy was performed. The authors noted absence of hepatocolic and splenocolic ligaments and lack of retroperitoneal fixation. Although pathology was negative for neoplasm, they theorized the lack of zygosis with persistent ascending and descending mesocolons helped to enable this presentation [3]. Furthermore, persistent descending mesocolons have been noted in previous reports as the etiology of colonic volvulus [8,9] and internal hernia [10].

Thus, two principle factors are causative in this case presentation of total ileocolic intussusception with rectal prolapse. The first being the lead point pathology of the villous adenoma, and the second being the increased colonic mobility associated with lack of zygosis.

## Conclusions

Intussusception is an uncommon etiology of bowel obstruction in adults and can be attributed to benign and malignant pathologies. Despite advancements in diagnostic accuracy, a high index of suspicion and clinical acumen is required for timely diagnosis and therapy of this condition in adults. Total ileocolic intussusception with rectal prolapse, found at the end of the adult intussusception spectrum, may be predisposed by an embryological variant lacking zygosis. For the acute care surgeon who may encounter this rare surgical emergency, the diagnosis should be considered in the differential of a prolapsing rectal mass and be expeditiously managed to optimize patient outcomes. Assessing for the absence of zygosis should be an adjunct to the operative procedure as well.

## Consent

Written infromed consent was obtained from the patient for publication of this Case Report and any accompanying images. A copy of the written consent is available for review by the Editor-in-Chief of this journal.

## Competing interests

The authors do not have any financial or non-financial competing interests to declare.

## Authors’ contributions

Study concept and design: JF, OB & YK. Acquisition of data: JF, OB. Analysis of data: JF, OB & YK. Drafting of manuscript: JF. Critical revision of manuscript: JF, YK. Study supervision: YK. All authors read and approved the final manuscript.
